# The observation records from whale and dolphin watching inshore of Hualien, eastern Taiwan

**DOI:** 10.3897/BDJ.11.e109649

**Published:** 2023-09-28

**Authors:** Chieh-Hsi Hu, Hsin Yi Yu, Daphne Z. Hoh, Dong Liang Lin

**Affiliations:** 1 Kuroshio Ocean Education Foundation, Hualien County, Taiwan Kuroshio Ocean Education Foundation Hualien County Taiwan; 2 Taiwan Biodiversity Information Facility, Biodiversity Research Centre, Academia Sinica, Taipei, Taiwan Taiwan Biodiversity Information Facility, Biodiversity Research Centre, Academia Sinica Taipei Taiwan

**Keywords:** sighting data, citizen-science, whale-watching tour, cetacean

## Abstract

**Background:**

The eastern waters of Taiwan have been lacking baseline and research data for several years. This study was initiated by Kuroshio Ocean Education Foundation (KOEF) in collaboration with the Turumoan whale-watching company since 1998, collecting long-term ecological data for cetaceans in the inshore of Hualien, eastern Taiwan. This dataset includes 10,675 records of cetacean sightings from June 1998 to December 2021. Collection of cetacean sighting records was paused for one year in 2001 due to budgetary reasons. All of the sighting records were collected by whale-watching boat guides that were trained by KOEF. Following a standardised protocol, guides used a handheld GPS device and cetacean sighting record sheets to document information about the cetacean species identification, location, time, number of individuals, the presence of mother-calf pairs and mixed-species groups and other states of each sighting during a whale-watching tour. The collection of citizen-science data during this period has significantly advanced Taiwan's cetacean baseline data in the study area. Additionally, we make data available to the public in the form of citizen-science, making a substantial contribution to the advancement of ocean scientific research. We have published the dataset on Global Biodiversity Information Facility, allowing users around the world to download the dataset.

**New information:**

This is currently the largest dataset of cetacean sighting records in Taiwan (last updated on 2023-09-05). We have also recorded several species on the International Union for Conservation of Nature (IUCN) Red List categorised as "Data Deficient" during our survey inshore of Hualien, eastern Taiwan, including Ginkgo-toothed beaked whales (*Mesoplodonginkgodens* Nishiwaki & Kamiya, 1958), Omura's whales (*Balaenopteraomurai* Wada, Oishi & Yamada, 2003) and killer whales (*Orcinusorca* (Linnaeus, 1758)). There are also sperm whales (*Physetermacrocephalus* Linnaeus, 1758), categorised as "Vulnerable" in the IUCN Red List and false killer whales (*Pseudorcacrassidens* (Owen, 1846)), categorised as "Near Threatened". This study is also the first and only long-term study that has documented cetaceans in the study area.

## Introduction

In 1978, the International Whaling Commission began tightening whaling restrictions and exerting pressure on Taiwan (Cheung 2023). In 1990, a non-governmental conservation organisation named Earth Trust filmed a dolphin-hunting event in Penghu, an outlying islet of Taiwan and subsequently screened the video in the United States. This incident caused substantial international pressure, leading to the protection of cetaceans in Taiwan in August of the same year and marked the beginning of increased research efforts on cetacean baseline data (Chou 2004). Due to the high cost of marine surveys, much of the ocean data collection today is done through Observation Platforms of Opportunity like ferries and whale-watching boats (Kiszka et al. 2007). The whale-watching industry in Taiwan began in 1997 and there are about 20 whale-watching companies today. Since 1998, we began collaborating with the Turumoan whale-watching company and each boat sent out for whale-watching was accompanied by Kuroshio Ocean Education Foundation (KOEF)'s guides. These guides are trained by cetacean experts on species identification, data collection and conducting outreach during the tour. In recent years, the cetacean sighting data have increased due to the growing participation of individuals in whale-watching activities and the increasing number of guides joining KOEF.

Cetaceans are marine mammals that play crucial roles in marine ecosystems with their diverse range of ecological functions, including being apex predators and serving as nutrient vectors. Cetaceans are vital to the functioning and stability of marine ecosystems, in which their species diversity and population status are essential indicators of overall marine health (Azzellino et al. 2014). Long-term monitoring data from citizen sources can be a straightforward tool for gaining a deeper understanding of biodiversity and how the growing human maritime activities are increasing the pressure on cetaceans (Azzellino et al. 2017, Coché et al. 2021, Global Biodiversity Information Facility 2023). According to past cetacean sighting and stranding records, there are approximately 30 species of cetaceans recorded in Taiwan (Chou 2004, Li et al. 2021). This dataset represents the first recorded long-term cetacean observation in Taiwan, which contains data on 20 different species (Fig. 1). By publishing this dataset (Yu and Hu 2023), we aim to assist research on richness, diversity, distribution and habitat preferences at different spatiotemporal scales, while minimising the need for duplicating research and speeding up scientific progress (European Commission 2012, Vinding et al. 2015, European Commission et al. 2020, Garcia-Cegarra et al. 2021, Gonzalez Garcia et al. 2022).

## Sampling methods

### Sampling description

**Figure 2.**
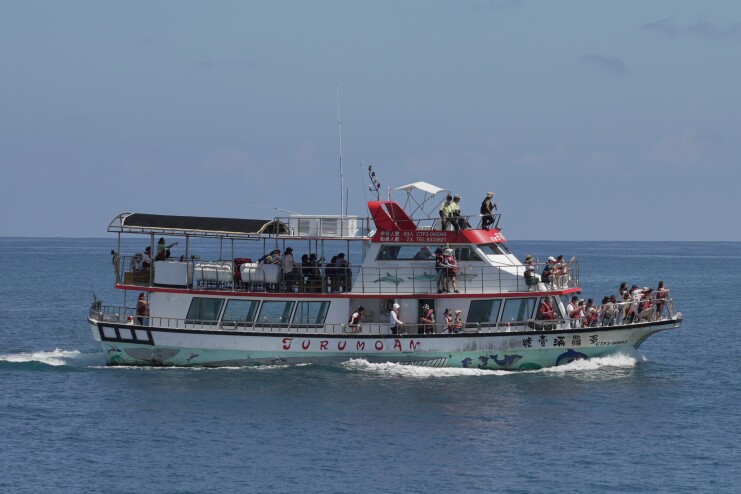
Whale-watching boat of the Turumoan whale-watching company, photo taken by Chieh-Hsi Hu.

For each whale-watching tour, each ship accommodates one guide trained by KOEF’s cetacean experts to record cetacean sightings (approximately 70% of our guides have three or more years of experience in detecting and identifying cetaceans, last updated on 2023-09-05). The boat follows a random path until cetaceans are detected and sometimes we also receive sighting reports from other whale-watching boats. While observing cetaceans, the guide documents the location and time of each sighting with a hand-held GPS device (Garmin GPSMAP 64st), identifies the cetacean species, estimates the number of individuals and confirms the presence of mother-calf pairs and mixed-species groups in the vicinity. Upon returning, the guide fills in the cetacean record sheet, which contains fields included in the Global Biodiversity Information Facility (GBIF) dataset. To avoid collecting duplicate sighting data, when there are trips with more than one vessel in the same area, only the data of one ship is recorded within the same trip. Trained volunteers subsequently input the data into a computer for digital storage. Most whale-watching tours are divided into five time periods: 6 am (on average 2 trips per month), 8 am (on average 8 trips per month), 10 am (on average 6 trips per month), 2 pm (on average 6 trips per month) and 4 pm (on average 4 trips per month), each trip is approximately 1.5 to 2.0 hours long; there are an average of 26 trips per month (Fig. 2).

### Quality control

Following electronic data entry, the records undergo a rigorous data cleaning process by a biologically-trained cetacean researcher of KOEF. All the scientific names of cetaceans are validated by the NomenMatch tool, which compares the names to the taxonomy backbones of GBIF, Catalogue of Life, Taiwan Catalogue of Life and Taiwan Biodiversity Network (Mai 2023) before they are added to the database. Geolocations are transformed into decimal degrees and verified by the Geographic Information System QGIS 3.10 (long-term release) software (QGIS.org 2019).

### Step description

**Citizen scientists' training**

KOEF's training for new guides is a 15-week course that combines onboard internships and practices with indoor training by cetacean experts to enhance citizen scientists' professional abilities to identify different cetacean species, estimate the cetacean group size, understand the definitions of the various terms on cetacean sighting record sheets and use the handheld GPS (Garmin GPSMAP 64st). Through two stages of evaluation, it ensures that all guides that pass the training possess sufficient skills for being citizen scientists and follow a standardised protocol.

**Data collection**

1. While leaving Hualien port, the guide records the departure time using a hand-held GPS (Garmin GPSMAP 64st).
2. The boat follows a random path until cetaceans are detected and sometimes we also receive sighting reports from other whale-watching boats. When cetaceans appear, the ship slowly approaches. The guide then marks the location and time with a hand-held GPS, identifies the cetacean species, estimates the number of individuals and confirms the presence of mother-calf pairs and mixed-species groups in the vicinity. The average speed of the ship is approximately 3-4 knots while it is close to the cetaceans and the average time the vessel stayed with each species is approximately 15 minutes.
3. When leaving the cetaceans, the guide marks the leaving time with a hand-held GPS. When entering the port, the guide uses the GPS to mark the arrival time. Apart from the spatiotemporal information, which is recorded through the hand-held GPS, the guide remembers all the other information until returning to the whale-watching company.
4. After returning to the whale-watching company, the guide fills in the cetacean record sheet, which contains fields included in the GBIF dataset.

**Data transcription**:

The cetacean record sheets are organised once a month and the information on the data sheets are entered into an Excel spreadsheet file by trained interns, volunteers and guides of KOEF.

**Open data preparation**

Before uploading data to GBIF, we use a Darwin Core quick reference guide (Darwin Core Maintenance Group 2021) to match each type of record in our dataset and convert each column's data following the Darwin Core Standard. After data formatting, the dataset is categorised into core and extension files. We use the GBIF data validator (Global Biodiversity Information Facility 2017) to determine potential issues and the data correcting process is completed and verified by a biologically-trained cetacean researcher of KOEF. If the identification of uncertain cetaceans lacks identification by description or photographic evidence, their species identification will be annotated as uncertain and will be excluded from this dataset.

## Geographic coverage

### Description

**Figure 3.**
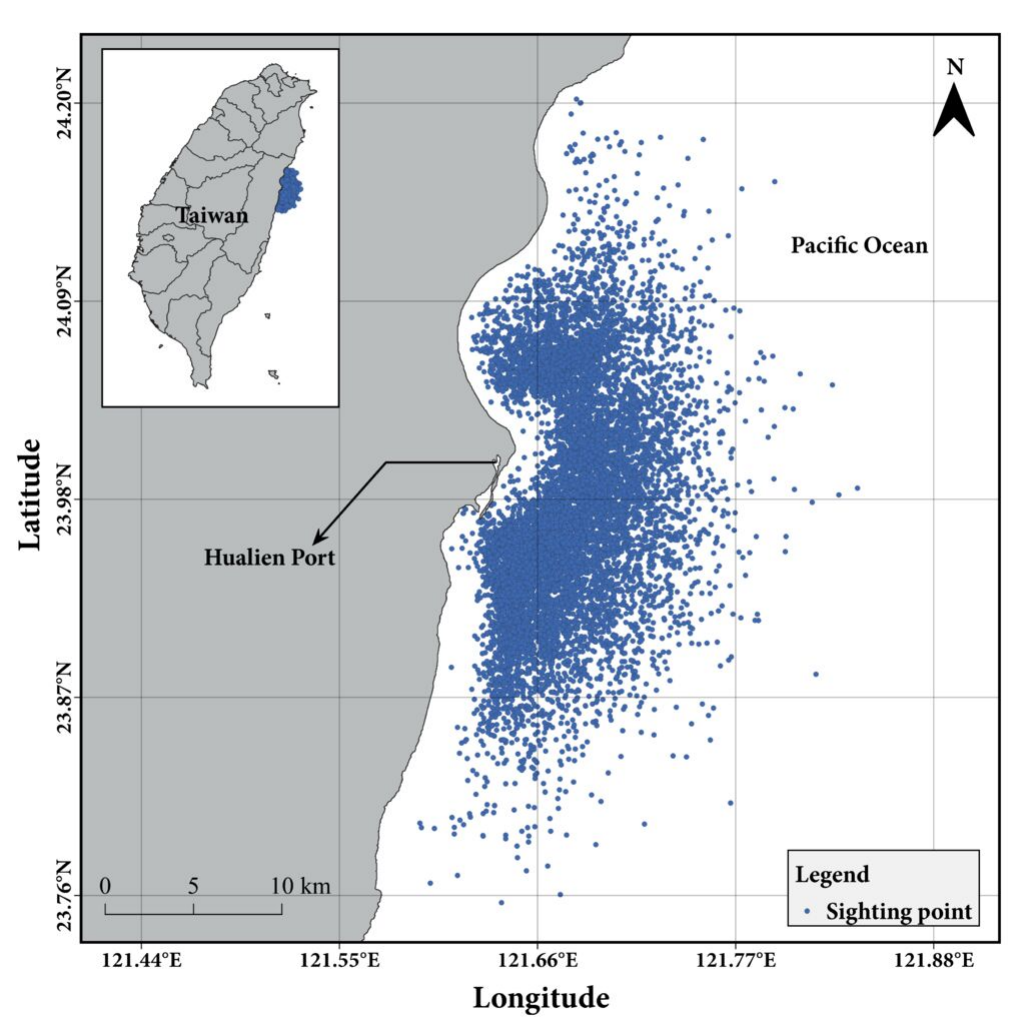
Sighting points of cetaceans inshore of Hualien, eastern Taiwan (n = 9,810; 91.9% of all sighting records are with coordinates). The map was plotted by using QGIS 3.10 (long-term release) software (QGIS.org 2019).

All of the sighting data were collected inshore of Hualien, eastern Taiwan (Fig. 3). Cetacean sighting records are all within an approximate range of 20 kilometres from Hualien Port, following the max speed of the vessels (10 to 12 knots) and the duration of whale-watching tours (1.5 to 2.0 hours).

### Coordinates

23.6° N and 24.3° N Latitude; 121.5° E and 121.9° E Longitude.

## Taxonomic coverage

### Description

**Figure 4.**
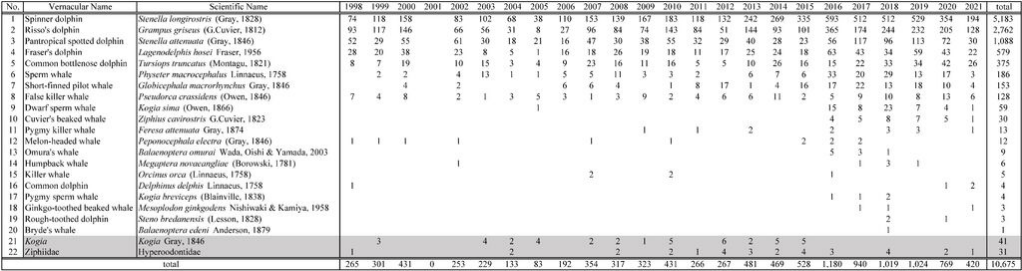
List of observed cetacean species in this dataset from 1998 to 2021. Prior to 2015, beaked whales were only recorded at the family level: Hyperoodontidae (Ziphiidae) and both dwarf sperm whale (*Kogiasima* (Owen, 1866)) and pygmy sperm whale (*Kogiabreviceps* (Blainville, 1838)) were mostly recorded at the genus level: *Kogia* Gray, 1846.

This dataset contains 10,675 cetacean sighting records covering 20 different species, from which 41 records were identified up to the genus level, which is *Kogia* Gray, 1846 and 31 records were identified up to the family level, which is Hyperoodontidae (Ziphiidae) (Fig. 4). In addition, six to 10 different species of cetaceans were observed each year, most of which were small to medium-sized odontocetes like the spinner dolphin (*Stenellalongirostris* (Gray, 1828)) and Risso's dolphin (*Grampusgriseus* (G.Cuvier, 1812)) (Fig. 5). This dataset has achieved a higher record number for some species. For instance, in the case of the spinner dolphin, this dataset currently has the highest number of records amongst all datasets in GBIF and, for Risso's dolphin, it ranks fourth (last updated on 2023-09-05).

### Taxa included

**Table:** 

Rank	Scientific Name	
kingdom	Animalia	
phylum	Chordata	
class	Mammalia	
order	Cetacea	
family	Delphinidae	
family	Hyperoodontidae	
family	Kogiidae	
family	Physeteridae	
family	Balaenopteridae	
genus	*Stenella*	
genus	*Grampus*	
genus	*Lagenodelphis*	
genus	*Tursiops*	
genus	*Pseudorca*	
genus	*Delphinus*	
genus	*Peponocephala*	
genus	*Kogia*	
genus	*Physeter*	
genus	*Globicephala*	
genus	*Megaptera*	
genus	*Orcinus*	
genus	*Feresa*	
genus	*Balaenoptera*	
genus	*Ziphius*	
genus	*Mesoplodon*	
genus	*Steno*	

## Temporal coverage

**Data range:** 1998-6-13 – 2021-12-12.

### Notes

**Figure 6.**
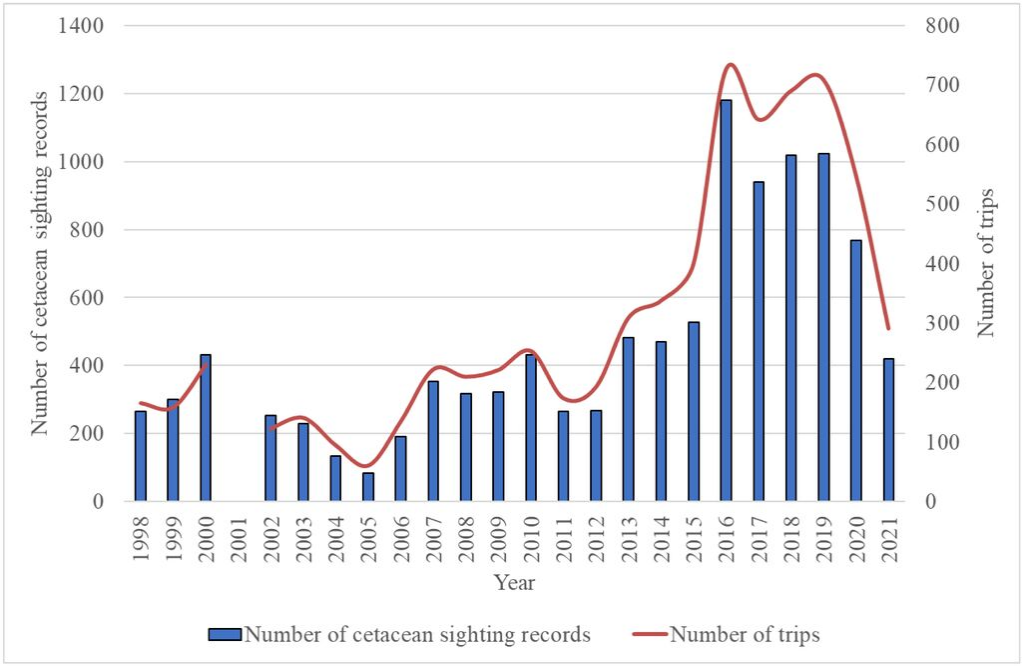
Number of cetacean sighting records and trips across 1998 to 2021; due to budgetary reasons, cetacean sighting records were suspended for one year in 2001.

KOEF was established in April 1998 and began collecting citizen-science data in collaboration with the Turumoan whale-watching company in June of the same year. Due to budgetary reasons, cetacean sighting records were suspended for one year in 2001 (Fig. 6). Due to the growing participation of individuals in whale-watching activities, both the number of trips and sighting records reached a peak between 2016 and 2020, but in 2021, the number of sighting records decreased significantly to around 400 due to the impact of the COVID-19 pandemic on loss of tourism.

## Usage licence

### Usage licence

Other

### IP rights notes

This dataset in the current work is licensed under a Creative Commons Attribution (CC-BY) 4.0 License. Any image materials in this data paper are licensed under the Creative Commons Attribution (CC-BY-NC) 4.0 License.

## Data resources

### Data package title

The observation records from whale and dolphin watching inshore of Hualien, eastern Taiwan

### Resource link

https://doi.org/10.15468/rg87xx

### Alternative identifiers

70e66bc4-a791-44c5-9c9b-1aa32934a909, https://ipt.taibif.tw/resource?r=koef_whale_dolphin_observation

### Number of data sets

3

### Data set 1.

#### Data set name

Event

#### Data format

Darwin Core standard (DwC)

#### Data format version

2022-02-02

#### Description

The event file is the core file of this dataset, which contains the time and location information for each sighting.

**Data set 1.** 

Column label	Column description
eventID	A unique identifier for the recording of a single cetacean sighting event.
eventDate	The sighting date-time of the occurrence. The time in eventDate means the start of the cetacean observation.
sampleSizeValue	The duration of each cetacean sighting event in minutes.
sampleSizeUnit	The time unit for the duration of each cetacean sighting event.
decimalLatitude	The geographic latitude (in decimal degrees, using the spatial reference system given in geodeticDatum) of the geographic centre of a Location. The location is based on the start of the cetacean observation.
decimalLongitude	The geographic longitude (in decimal degrees, using the spatial reference system given in geodeticDatum) of the geographic centere of a Location. The location is based on the start of the cetacean observation.
geodeticDatum	The ellipsoid, geodetic datum or spatial reference system (SRS) upon which the geographic coordinates given in decimalLatitude and decimalLongitude were based.
country	The name of the country or major administrative unit in which the Location occurs.
countryCode	The standard code for the country in which the Location occurs.
locality	Less specific geographic information is provided in this column. Events with no geographic coordinates are recorded in general terms as "inshore of Hualien".

### Data set 2.

#### Data set name

Occurrence

#### Data format

Darwin Core standard (DwC)

#### Data format version

2022-02-02

#### Description

Occurrence is an extension file of this dataset that includes Detailed information about species sighted. The records with occurrenceStatus as "absent" are not included in this dataset.

**Data set 2.** 

Column label	Column description
eventID	A unique identifier for the recording of a single cetacean sighting event.
type	The nature or genre of the resource.
basisOfRecord	The specific nature of the data record.
occurrenceID	An identifier for the Occurrence (as opposed to a particular digital record of the occurrence).
eventDate	The sighting date-time of the occurrence. The time in eventDate means the start of the cetacean observation.
occurrenceStatus	A statement about the presence or absence of a Taxon at a Location.
scientificName	The full scientific name.
kingdom	The full scientific name of the kingdom in which the taxon is classified.
phylum	The full scientific name of the phylum or division in which the taxon is classified.
class	The full scientific name of the class in which the taxon is classified.
order	The full scientific name of the order in which the taxon is classified.
family	The full scientific name of the family in which the taxon is classified.
genus	The full scientific name of the genus in which the taxon is classified.
taxonRank	The taxonomic rank of the most specific name in the scientificName.
vernacularName	A common or vernacular name.
individualCount	The number of individuals present at the time of the Occurrence.

### Data set 3.

#### Data set name

MeasurementOrFacts

#### Data format

Darwin Core standard (DwC)

#### Data format version

2022-02-02

#### Description

MeasurementOrFact is an extension file of this dataset that contains data that are not defined by the Darwin Core standard (DwC), but present in the cetacean sighting records. For details of MeasurementOrFact field items and corresponding data descriptions, see Suppl. material 1.

**Data set 3.** 

Column label	Column description
eventID	A unique identifier for the recording of a single cetacean sighting event.
measurementID	An identifier for the MeasurementOrFact (information pertaining to measurements, facts, characteristics or assertions). May be a global unique identifier or an identifier specific to the dataset.
measurementType	The nature of the measurement, fact, characteristic or assertion. See Suppl. Material 1 for a detailed description of each field.
measurementValue	The value of the measurement, fact, characteristic or assertion.
measurementMethod	A description of, or reference to (publication, URI), the method or protocol used to determine the measurement, fact, characteristic or assertion.

## Additional information

To provide users of this dataset with a better understanding of its details, this section shows some limitations and additional information associated with the use of this dataset:

1. Cetacean sightings may be recorded repeatedly during different time slots within various trips on the same day.
2. Cetaceans in mixed-species groups will have their own separate sighting records. For example, when Risso's dolphins (*Grampusgriseus* (G.Cuvier, 1812)) mix with Fraser's dolphins (*Lagenodelphishosei* Fraser, 1956), there will be two entries: one for Risso's dolphins mixed with Fraser's dolphins and another for Fraser's dolphins mixed with Risso's dolphins. Both records will be documented according to the data included in the GBIF dataset.
3. The reason why cetacean sighting data is primarily collected during the spring and summer (March to August) is that the sea conditions around the inshore of Hualien are often better in these two seasons. This allows for more opportunities to collect data; whale-watching boats are often suspended in autumn and winter (September to February) due to the northeast monsoon, so the amount of data collected is much less than in spring and summer.
4. Since 2016, the Turumoan whale-watching company has adjusted the time of whale-watching tours from the previous 2 to 2.5 hours to 1.5 to 2 hours; therefore, the geographic coverage of cetacean sighting records became smaller after 2016 compared with the data collected in earlier years.

## Supplementary Material

08C720ED-0460-5783-94E2-C356E83E846E 10.3897/BDJ.11.e109649.suppl1 Supplementary material 1 MeasurementOrFact field items and data descriptions Data type biological Brief description MeasurementOrFact field items and corresponding data descriptions for this dataset. File: oo_902551.txt https://binary.pensoft.net/file/902551 Chieh-Hsi Hu

## Figures and Tables

**Figure 1a.**
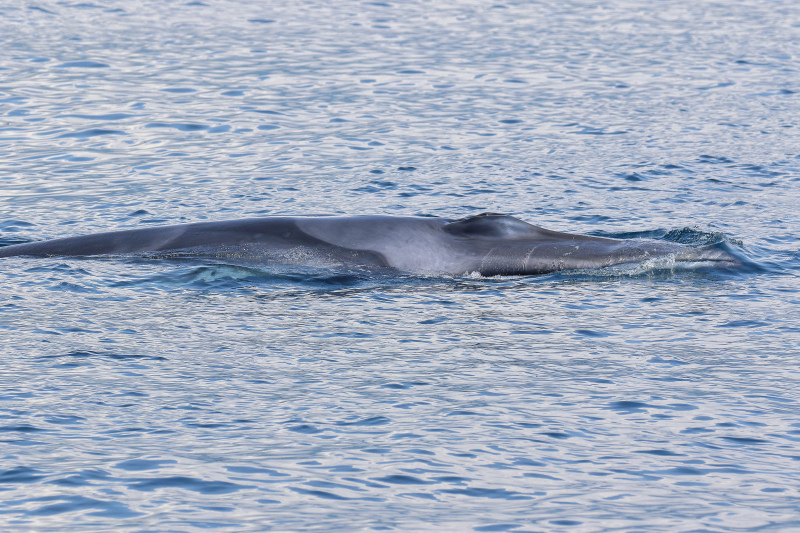
Omura's whale (*Balaenopteraomurai* Wada, Oishi & Yamada, 2003), "Data Deficient", photo taken by Ray Chin.

**Figure 1b.**
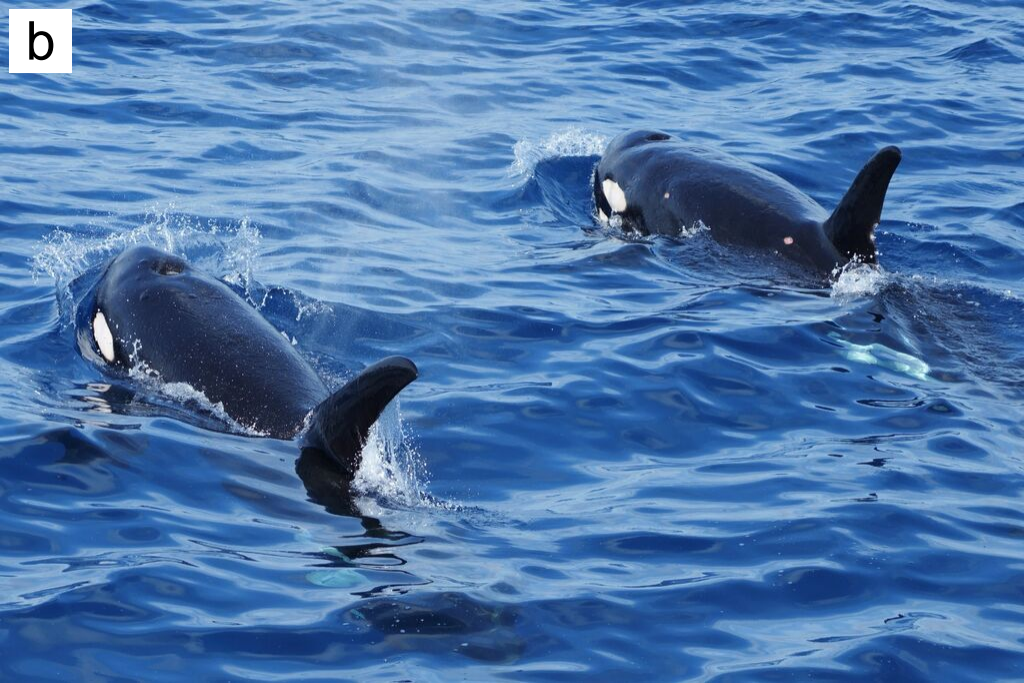
Killer whales (*Orcinusorca* (Linnaeus, 1758)), "Data Deficient", photo taken by Chieh-Hsi Hu.

**Figure 1c.**
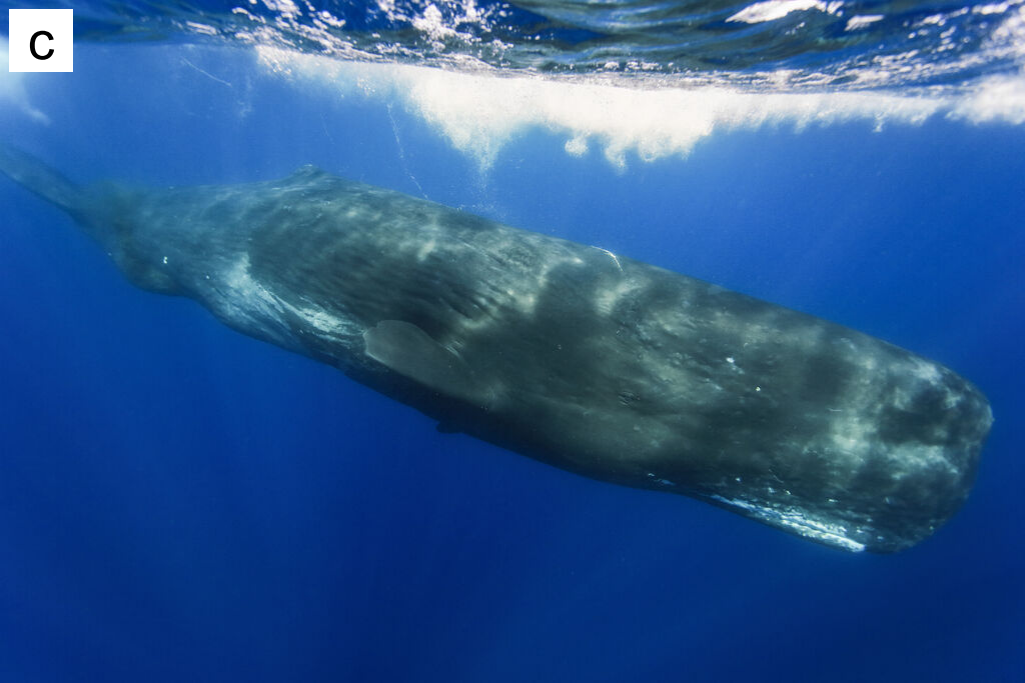
Sperm whale (*Physetermacrocephalus* Linnaeus, 1758), "Vulnerable", photo taken by Ray Chin.

**Figure 1d.**
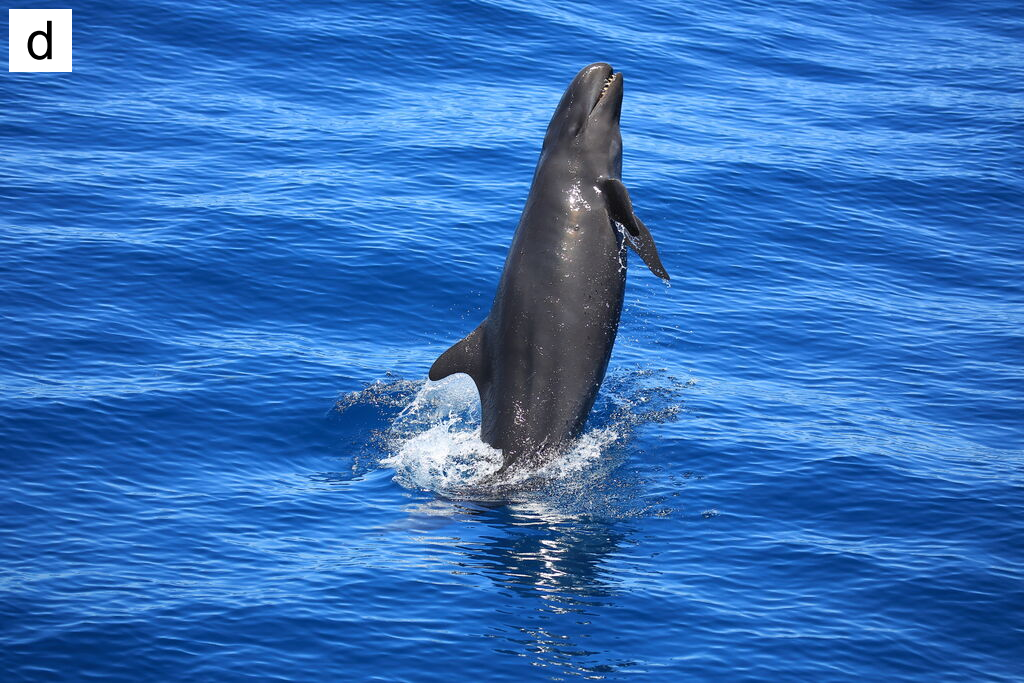
False killer whale (*Pseudorcacrassidens* (Owen, 1846)), "Near Threatened", photo taken by Captain Wen-Lung Jiang.

**Figure 5a.**
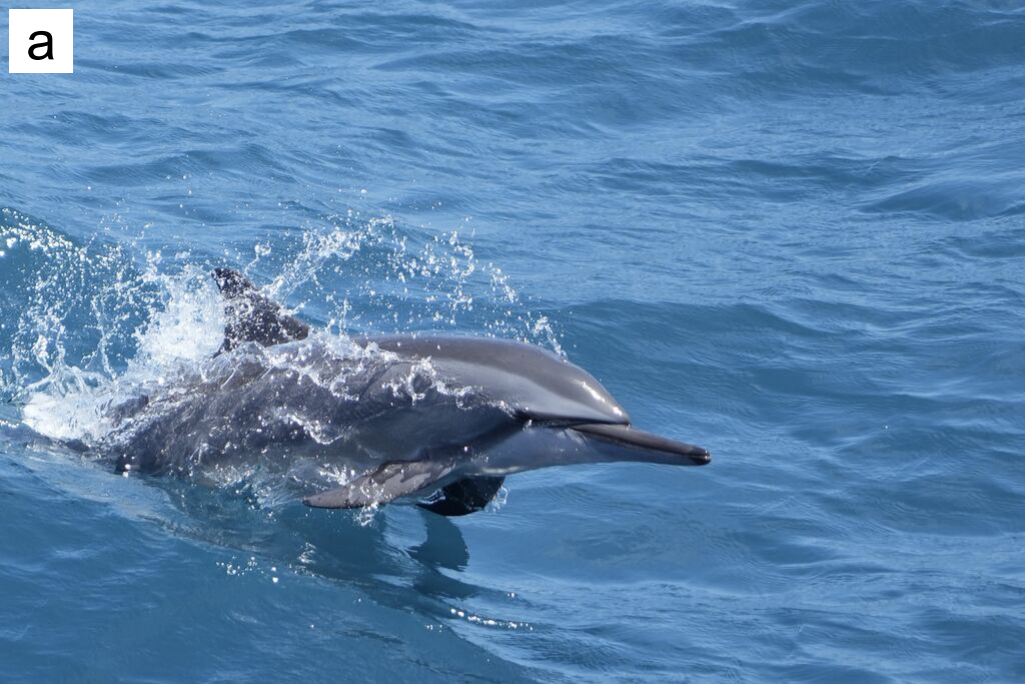
Spinner dolphin (*Stenellalongirostris* (Gray, 1828)), photo taken by Chieh-Hsi Hu.

**Figure 5b.**
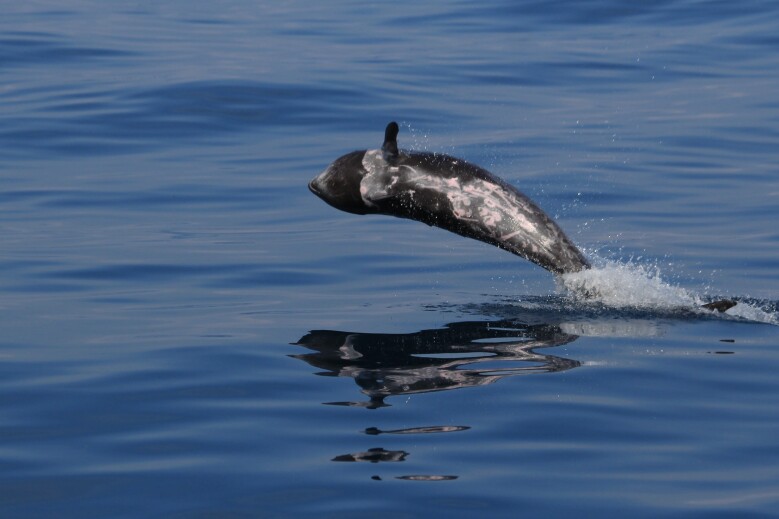
Risso's dolphin (*Grampusgriseus* (G.Cuvier, 1812)), photo taken by Captain Wen-Lung Jiang.
